# Killing Neck Snares Are Inhumane and Non-Selective, and Should Be Banned

**DOI:** 10.3390/ani15152220

**Published:** 2025-07-28

**Authors:** Gilbert Proulx

**Affiliations:** Alpha Wildlife Research & Management, 229 Lilac Terrace, Sherwood Park, AB T8H 1W3, Canada; gproulx@alphawildlife.ca

**Keywords:** animal welfare, fur-trapping, humaneness, International Humane Trapping Standards, killing neck snares, selectivity, non-target captures

## Abstract

In North America, where fur trapping remains an active practice, killing neck snares continue to be used for capturing canids, particularly red fox (*Vulpes vulpes*), coyote (*Canis latrans*), and gray wolf (*Canis lupus*). However, over the last 50 years, scientific studies have consistently demonstrated that killing neck snares are inhumane—meaning that snared animals do not lose consciousness within five minutes and may remain alive for hours—and non-selective, often capturing a wide range of non-target wild and domestic animals. This non-selectivity can contribute to the local extirpation of certain species. The continued use of killing neck snares reflects a disregard for the welfare of wild mammals and poses risks to the sustainability of their populations. This persistence appears to be driven by misinformation, widespread myths, and inadequately implemented international trapping standards. These issues lead to critical questions about what must be performed to prohibit the use of these devices.

## Simple Summary

This paper reviews scientific evidence gathered in the last 50 years showing that both manual and power killing neck snares are inhumane, i.e., they do not quickly render captured animals unconscious, and are non-selective, i.e., they capture many non-target species. The paper explores why killing neck snares are still being used in spite of their shortcomings, and presents a series of questions regarding steps that should be taken to ban them.

## 1. Introduction

In North America, furbearer trapping is being practiced as a way of life, but more often as a recreational activity. It is employed to control nuisance animals or predators, “harvest” furs, glands, or skulls, or implement specific wildlife management programs [1]. However, it is a highly controversial and invasive activity that has drawn sustained criticism from both the public and the scientific community since the early 1900s [2,3]. The continued use of unacceptable trapping devices and the retention of the ‘old ways’ by trappers and pest controllers are largely the causes of so much controversy in mammal trapping [1]. Case in point, despite significant technological improvements, many antiquated trapping systems are still being used today [4]. Killing neck snares are one example of such outdated trapping devices which have been repeatedly found to be inhumane and unselective [5], but are still popular today because they are cheap, lightweight, easy to set and camouflage (except in the case of power snares), efficient at capturing a diversity of furbearers, and claimed to be humane by trappers [5,6,7].

In the last decade, environmental and animal welfare groups have increasingly called for the banning of killing neck snares [8,9,10,11,12]. Nonetheless, killing neck snares are still being used for the capture of furbearers in Canada and the United States. In this paper, I review (1) arguments presented against the utilization of killing neck snares, namely, humaneness and non-selectivity; (2) myths and misinformation presented by people advocating their use; and (3) standards and accepted practices legitimizing the use of killing neck snares in Canada. Finally, I pose critical questions about what actions are necessary to bring about a ban on killing neck snares in the near future.

## 2. Description of Killing Neck Snares

**Figure 1:**
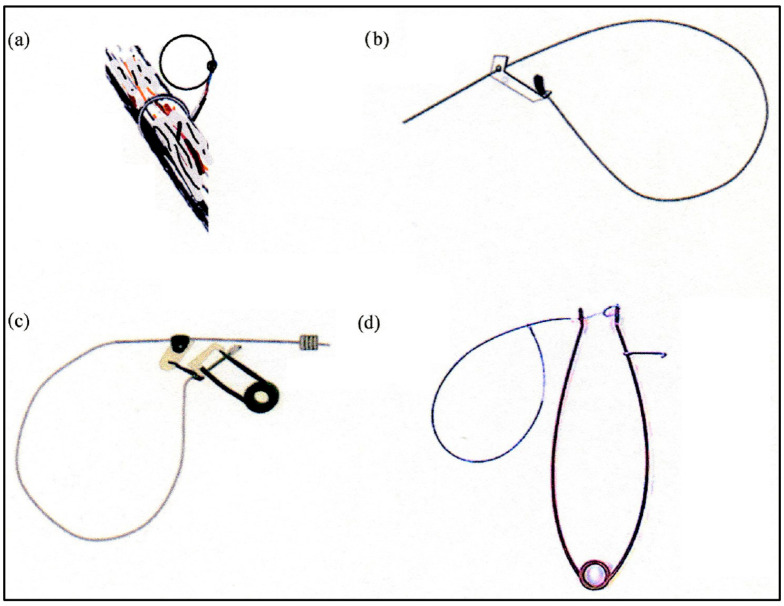
Killing neck snares: (**a**) manual snare, brass wire snare for red squirrels; (**b**) manual snare, braided wire with a one-way lock; (**c**) manual snare, braided wire with a Cam-Lock and a compression spring; and (**d**) power snare, braided wire. Snares b, c, and d are used for the capture of canids.

Manual snare—Brass or stainless steel wires are used to snare small mammals (e.g., snowshoe hare *Lepus americanus*, red squirrel *Tamiasciurus hudsonicus*) (Figure 1). Snares for mesocarnivores and large mammals usually are made of a braided, galvanized, or stainless steel wire (diameter: 1/16 to 1/8 inch—1.6 to 3.2 mm) with a loop (Figure 1). The cable has a one-way locking tab that only allows the noose to close and stops it from re-opening [13]. In this design, the animal itself provides the energy necessary to tighten the noose.

Power killing neck snares—These devices use a spring to supply the energy required to tighten the noose around the animal’s neck (Figure 1). Locks are not needed because the clamping force is delivered by the spring pulling on the snare wire [5].

Snare sets—Snares are placed along animal trails or in enclosed areas baited with lures or food. In the case of gray wolf (*Canis lupus*) trapping, as many as ten or more killing neck snares may be set around a large bait (“saturation snaring”) to capture most members of a pack [5].

## 3. Scientific Evidence

### 3.1. Animal Welfare Standards for Humaneness

The Agreement on International Humane Trapping Standards (AIHTS) considers that a trap is humane if it renders small furbearers such as martens (*Martes* spp.) irreversibly unconscious in ≤3 min, and larger animals such as canids in ≤5 min [14,15,16].

The Agreement on International Humane Trapping Standards also considers that traps are acceptable if at least 80% of the animals lose consciousness within the specific time limits of 3 or 5 min, depending on the animal species [15].

It is noteworthy to mention that in past scientific assessments of traps, ≥70% of captured animals had to become unconscious within specific time periods [13]. Using the approximation to the binomial distribution, a snare with a ≥70% performance would need to render 9/9 animals irreversibly unconscious within 3 min. When developing new international trapping standards, my colleagues and I considered that a minimum performance level of 85% would be realistic [17]. To obtain an estimated 85% performance, 13/13 animals would have to lose consciousness within the time limit [17].

Research programs that assessed the ability of killing neck snares to quickly kill furbearers repeatedly demonstrated that snared animals remained conscious for long periods of time, exceeding the 5-min time limits accepted by the international trapping standards for large furbearers (Table 1).

When I assessed the performance of killing neck snares for gray wolves and coyotes (*Canis latrans*) on a trapline, I found that a coyote lost consciousness 14 h 16 min after being snared, and a wolf, 3 h 39 min after capture [13]. On the basis of the approximation to the binomial distribution, the killing snares would not render ≥70% of canids irreversibly unconscious within 5 min. Therefore, the performance of the killing neck snares would fail to meet the animal welfare criteria of the international animal welfare trapping standards. Consequently, I concluded that no more assessments were required to demonstrate that killing neck snares were not humane for the capture of these canids [13].

It is worth mentioning that the assessment of killing devices, such as snares, should take into consideration that animals may break the snare cable and escape. When animals escape with a snare tightened around their neck, they are subject to debilitation, infection, and starvation [13,18,19,20]. They experience a long and painful death [5].

In conclusion, studies conducted in laboratory and in the field (Table 1) have shown that killing neck snares did not meet the criteria of the Agreement on International Humane Trapping Standards, and are therefore inhumane.

### 3.2. Non-Selectivity

Killing neck snares are not selective, and they capture many wild and domestic non-target species. Snares capture mammals of all sizes, and include many carnivore species which are attracted to baits, and ungulates and other species which travel on trails where snares are set (Table 2). The capture of non-target species can result in the extirpation of some species [18], and the removal of species at risk such as wolverine (*Gulo gulo*) and woodland caribou (*Rangifer tarandus*) [5,21]. In the foothills of Alberta and traplines adjacent to Banff and Jasper National Parks, killing neck snares set to capture gray wolves remove 8–12% of the resident mountain lion (*Puma concolor*) population each year [22,23]. These deaths are likely additive to other losses [24] and could jeopardize the persistence of mountain lion populations in the Canadian Rockies [23].

In Canada, from 2010 to 2020, a minimum of 162 dogs (*Canis familiaris*) and cats (*Felis catus*) were captured in killing neck snares set for canids in suburban areas [25]. The capture of domestic animals in killing neck snares has also been reported outside North America (Table 2) [26,27].

**Table 1:** Summary of published research that assessed the ability of killing neck snares to quickly render furbearers irreversibly unconscious.

Snare Type	Species	Methods and Findings	Reference
Braided wire manual snare	Coyotes (*Canis latrans*) in the wild.	Snares were set along a 27 km route within a 15.5 km^2^ area. They were checked daily except during rainy weather. The trapping effort was 20,436 snare-days, where a snare-day was one snare operative for 24 h. Of 65 coyotes snared in this study, 59% were neck catches, 20% flanks, 11% front leg and neck, and 10% foot. Of the catch, 52% were dead in the morning after being snared, and 48% were alive. The authors concluded that snares are less humane than other predator control tools.	[18]
Braided wire manual snare	Anesthetized red foxes (*Vulpes vulpes*) in laboratory conditions, and one free-ranging fox in a compound.	The study used snare wire diameters and techniques recommended by experienced snare trappers. Experimentation was conducted using anesthetized animals and snares with locks. The objective was to determine the time to death of red foxes snared in the neck region. Researchers applied force to tighten the noose to its smallest diameter, but animals were still breathing 30–40 min after snaring. The length of time elapsing before loss of consciousness and brain death was excessive in most tests. Necropsy findings showed that 8 of 18 foxes exhibited varying degrees of pulmonary edema. A free-ranging fox captured in a snare set in a compound fought the snare deliberately and consistently, and was subjected to euthanasia after five minutes. Researchers believed that the fox could have remained in the snare alive for an extended period of time. Researchers concluded that manual snares could not offer a potentially humane death for canids.	[14]
Brass wire manual snare	Red squirrels (*Tamiasciurus hudsonicus*) in laboratory and in simulated environment.	Controlled field tests required that snared red squirrels lose consciousness within 3 min. Two squirrels in simulated environments died or were euthanized 4 min after being captured. In subsequent tests in simulated environments, three red squirrels were still conscious after 3 min and were euthanized. Researchers concluded that snaring does not offer a suitable means of trapping red squirrels humanely.	[14]
3 types of power snares with braided wire	Red foxes in semi-natural environments.	The study was conducted in a 2.2 ha forested compound. Tests included three types of power snares, powered by one or two torsion springs to tighten the noose around an animal’s neck. Cable sizes were 1.2 or 1.6 mm in diameter. Tests required that captured animals lose consciousness within 5 min. Between 50% and 100% of the animals did not lose consciousness within 5 min, and most of them were euthanized. Researchers concluded that power snares developed to quickly kill large furbearers appear to have limited application in the search for humane trapping methods.	[28]
Stainless steel wire manual snare	Snowshoe hare (*Lepus americanus* in semi-natural environments	The study was conducted in a 2.2 ha forested compound. All snowshoe hares were allowed a minimum of 3 days to acclimate to the simulated natural environment before any tests were conducted. A 0.02 gauge stainless steel wire was used. Tests with nine animals showed that the sum of exerting escape attempts lasted, on average, 2.5 min (SE = 0.4). On average, the time to confirmed death was 18 min (SE = 4.4) after capture of the animals.	[29]
3 types of manual snares with braided galvanized aircraft cable	Coyotes in the wild.	In winter predator control programs in Montana, out of 374 captures, 301 (89%) coyotes were snared by the neck. Nearly 50% of the animals were still alive or had escaped the morning after being snared. More than 20% were still alive in one snare type.	[30]
Manual snare with braided wire	Canids.	Injuries caused by killing neck snares are described and compared to those caused by steel-jawed leghold traps. Canids are not always captured by the neck, and they suffer severe injuries similar to those observed in animals captured in steel-jawed leghold traps. Abdominal captures may even lead to disembowelment. Neck-captured animals, which do not die rapidly, develop extreme swelling of the neck, head, and eyes, which may freeze shut in winter.	[31]
Manual snare with braided wire	1 coyote and 1 gray wolf (*Canis lupus*) on a trapline in the wild.	This study occurred on a trapline in the backcountry of Alberta. A trapper had set several snares made of 0.24 cm in diameter, 2.5 m long, aircraft galvanized steel cables. Snares were fastened to rebar anchors, and they were all equipped with one-way Cam-Locks. Snare loops were 30 cm in diameter, and they were set about 30 cm from the ground, more than 10 m from a bait station, which consisted of body parts of deer (*Odoileus* spp.) and other animals. I set and camouflaged six cameras, at least 1.5 m above the ground and at least 4 m away from snares. Cameras were programmed for 20 to 30 s-long videos, with a 20 s delay between motion-triggered recordings. I returned to the trapping site a few days later, after a few centimeters of snow had fallen. Snares had captured 1 coyote and 1 gray wolf. I reviewed the recordings and noted capture time and irreversible loss of consciousness based on the loss of corneal reflex. During daylight, the blinking of the eyelids indicated that the animals were alive. During nighttime, interruption of the eyeshine (reflection of the camera light from the tapetum lucidum of the eyes) due to the blinking of the eyelids confirmed that the animals were still conscious. The coyote lost consciousness 14 h 16 min after being captured. The wolf lost consciousness 3 h 39 min after being captured. During nearly 50% of their respective capture periods, both animals constantly struggled and showed signs of distress. On the basis of the approximation to the binomial distribution, where 9/9 animals must lose consciousness within 5 min (time limit in the AIHTS), these findings showed that killing neck snares could not humanely kill ≥ 70% of captured canids.	[13]
Unspecified	37 wolves, a protected species in Poland.	Review of 37 wolves snared in Poland between 2014 and 2020, and monitoring of 16 wolves with radio collars and camera traps. Researchers reported evidence of old and severe injuries caused by previous snaring, as well as recordings from camera traps revealing that some wolves escaped from snares and live severely disabled and alone, or supported by their pack mates.	[20]

**Table 2:** Non-exhaustive list of studies reporting the capture of non-target species in killing neck snares.

Target Species	Non-Target Captures	Reference
Coyote (*Canis latrans*)	Bobcats (*Lynx rufus*); American badger (*Taxidea taxus*); northern raccoon (*Procyon lotor*); striped skunk (*Mephitis mephitis*); gray fox (*Urocyon cinereoargenteus*); peccary (*Pecari tajacu*); cottontail (*Sylvilagus floridanus*); black-tailed jackrabbit (*Lepus californicus*); and armadillo (*Dasypus novemcinctus*); gopher tortoise (*Gopherus berlandieri*); and domestic animals.	[18]
Snowshoe hare (*Lepus americanus*)	American marten (*Martes americana*).	[32,33]
Gray wolf (*Canis lupus*)	Moose (*Alces americanus*), Sitka black-tailed deer (*Odoileus hemionus sitkensis*), and woodland caribou (*Rangifer tarandus*).	[34]
Gray wolf (*Canis lupus*)	Mountain lion (*Puma concolor*).	[22,23]
Red fox (*Vulpes vulpes*)	Stone martens (*Martes foina*), mongoose (*Herpestes ichneumon*), wild boar (*Sus srcofa)*, and dogs (*Canis familiaris*).	[27]
Coyote, gray wolf, and red fox	Specimens submitted to the Canadian Wildlife Health Cooperative from 1990–2014: American black bear (*Ursus americanus*); bobcat (*Lynx rufus*); Canada lynx (*Lynx canadensis*); fisher (*Pekania pennanti*); mountain lion (*Puma concolor*); snowshoe hare (*Lepus americanus*); white-tailed deer (*Odocoileus virginianus*); wolverine (*Gulo gulo*); bald eagle (*Haliaeetus leucocephalus*); barred owl (*Strix varia*); common raven (*Corvus corax*); golden eagle (*Aquila chrysaetos*); goshawk (*Accipiter gentilis*); great horned owl (*Bubo virginianus*); red-tailed hawk (*Buteo jamaicensis*); rough-legged hawk (*Buteo lagopus*).	[5]
Red fox and rabbit	In 2017–2021, of 505 snaring non-target captures attended by the RSPCA: 72 European badgers (*Meles meles*); 17 unspecified deer; 5 gray squirrels (*Sciurus carolinensis*); 3 brown hares (*Lepus europaeus*); 3 hedgehogs (*Erinaceus europaeus*); 3 muntjacs (*Muntiacus reevesi*); 123 cats (*Felis catus*), 21 dogs (*Canis familiaris*), 2 horses (*Equus ferus caballus*), 2 sheep (*Ovis aries*), 1 cow (*Bos taurus*); 1 blackbird (*Turdus merula*), 1 buzzard (*Buteo buteo*); 1 coot (*Fulica* spp.); 10 feral pigeons (*Columba livia domestica*), 7 mute swans (*Cygnus olor*), 3 Canada geese (*Branta canadensis*), 3 grey herons (*Ardea cinerea*), 1 chicken (*Gallus gallus*), 1 greylag goose (*Anser anser*), 1 kestrel (*Falco* spp.), 1 magpie (*Pica pica*), 1 pheasant (*Phasianus* spp.), 1 wood pigeon (*Columba palumbus*), and 1 domestic duck (*Anas platyrhynchos*).	[26]

### 3.3. History Repeating Itself

Scientific studies conducted over the last 50 years in North America have shown that killing neck snares are neither humane nor selective (Table 1 and Table 2). They significantly impact the welfare of individual animals and the persistence of populations. Clearly, the use of killing neck snares disregards the World Organization for Animal Health’s definition of animal welfare, as they profoundly affect the physical and mental state of captured animals [35]. They also fail to meet the Canadian Council on Animal Care’s Wildlife Guidelines, which stipulate that “every effort must be made to minimize stress to avoid distress of the captured animal” [36]. Since it is well established that killing neck snares are inhumane and affect the welfare of many non-target species, one may wonder what justification trappers offer for their continued use.

### 3.4. Myths and Misinformation

Myth 1

The general discourse—Trappers commonly believe that snared animals die of asphyxiation.

Counter evidence—Researchers from the Federal Provincial Committee for Humane Trapping failed to render anesthetized red foxes unconscious with manual snares, even after tightening the snare to 2–3 cm less than the diameter of an animal’s neck [14]. Researchers were able to push a swab into the trachea of animals while the snare was still tight. When testing power killing neck snares, a veterinarian noted that, although a 2 mm probe could not be passed down the trachea of 2 foxes, good aeration was present in the inflated lungs of each animal as evidenced by the organ’s pinkish/red color [37]. The fact is that it is difficult to constrict the trachea of a fox, a gray wolf, or a coyote because of its rigid cartilaginous rings and adjacent musculature [13].

Myth 2

The general discourse—The past president of the Alberta Trappers’ Association claims that “*snares aren’t designed to choke off the air supply like many suppose. They are designed to close off the carotid arteries*” [7].

Counter evidence—Due to collateral blood circulation, it is difficult, if not impossible, to stop blood flow to and from the brain by tightening a snare on the neck [38]. For example, researchers reported the case of a 2-year-old male coyote found in a moribund state on Prince Edward Island, Canada [19]. The killing neck snare had cut through the soft tissues of the neck, transecting the full diameter of the trachea, and was embedded in scar tissue between the trachea and the esophagus. The animal was alive, although the snare had completely obstructed both jugular veins and both common carotid arteries [19]. Similarly, researchers from the Federal Provincial Committee for Humane Trapping could not fully occlude the carotid arteries, even after tightening the snare to its maximum [14].

Myth 3

The general discourse—Trappers often report that killing snares render animals unconscious within seconds, suggesting they are among the most humane trapping devices. For instance, a trapper featured in Pyramid Productions’ *Unnatural Enemies: The War on Wolves* claimed that a killing snare tightens on a wolf’s neck and renders it unconscious in seconds [39].

Counter evidence—No scientific study ever reported that killing neck snares could render canids unconscious in seconds (Table 1). In all my years of research on snares and during 50 years of field work on mammals and capture techniques, I have never seen this outcome.

Myth 4

The general discourse—Trappers claim that new snaring technology, which includes a small torsion spring on the loop, applies extra pressure to the cable lock [40].

Counter evidence—This modification was never scientifically tested, and no published studies ever reported that the addition of a small torsion spring to the snare loop increased the constriction force of the snare to produce a humane death. While power snares use stronger torsion springs that exert constant high pressure, even these failed to render red foxes unconscious within five minutes [28]—the time threshold required by the Agreement on International Humane Trapping Standards for certifying traps for large animals [15].

Myth 5

The general discourse—Trappers claim that killing neck snares can be selective [41,42].

Counter evidence—Selectivity can be marginally improved by adjusting loop size and height, adding breakaway devices to allow some species to escape, and using diverter wires that deflect the snare when contacted by the muzzles of ungulates [13]. Baits may also be concealed to avoid attracting birds of prey. However, carnivores possess a highly developed sense of smell [21,43,44] and will investigate any bait placed behind a snare. Consequently, wolverines, fishers (*Pekania pennanti*), mountain lions, and other non-target species are at risk of being caught. Any animal using a trail where a snare is set may be captured. As numerous studies have shown [5,18,26], it is a fallacy to claim that killing neck snares selectively capture only target species.

Myth 6

The general discourse—Trappers assert that modern snares are the only legal and humane methods available for the lethal harvest and control of wild canids [45].

Counter evidence—In reality, killing neck snares are designed to restrain an animal until it dies from pulmonary congestion and exhaustion after prolonged struggle [13]. These are not true killing devices, and despite claims of improved technology, they remain inhumane. Thus, killing neck snares are unacceptable restraining devices and could be replaced by better alternatives. Thirty years ago, studies demonstrated that rubber-padded leghold traps significantly reduced the frequency of serious injuries [46,47]. With short trap-checking intervals, animals experience less distress and can be humanely dispatched by trappers [48]. However, even padded leghold traps do not eliminate all injuries—animals may suffer tooth damage, exhaustion, and distress [49]. Tranquilizer tabs, which release a drug when bitten, can be added to the jaws of traps [50], reducing struggling and injury severity [51,52].

### 3.5. Poor International Trapping Standards

In 1995, under pressure from the Council of the European Union (EU), negotiations began with fur-producing countries to develop the Agreement on International Humane Trapping Standards. The goal was to ban steel-jawed leghold traps in signatory countries and certify traps that address animal welfare concerns [3].

The standards of the Agreement have been subject to recurring criticism by scientists [3,53,54,55,56]. The main reason is that these standards are fur trade-oriented standards, which are not representative of state-of-the-art trapping technology, and fail to properly address the welfare of captured animals [3]. Nevertheless, this agreement has been adopted by governments without question, and it is implemented throughout Canada for the management of fur trapping.

In their review of the implementation of the Agreement on International Humane Trapping Standards, Feldstein and Proulx [4] explained that killing neck snares can be used in Canada because they are exempt by a “competent authority”, and they are considered homemade devices [15]. Also, although the agreement requires that trapping methods must be tested to demonstrate their conformity with the standards within 5 years of the entry into force of the Agreement in 1999, permission may be given on an interim basis only while research continues to identify replacement traps. No replacement traps for killing neck snares have ever been developed since the enforcement of the standards in Canada [4]. Trappers using snares have apparently benefited from an interim permission that has already lasted more than two decades. The Agreement on International Humane Trapping Standards actually ensures that inhumane but popular killing neck snares will continue to be used in Canada [4].

In the United States, there are no national trapping standards. Instead, jurisdictions rely on Best Management Practices (BMPs), which in practice amount to approving whatever devices are commonly used by trappers [13]. BMP research programs have been found inadequate to properly assess trapping systems [57], and there is no research or development program for killing neck snares.

## 4. Discussion

Scientific assessments have shown that killing neck snares are unable to render captured animals irreversibly unconscious within a few minutes or to kill them quickly. Studies have also demonstrated that these snares cannot selectively capture target animals. Killing neck snares consistently failed to meet today’s animal welfare standards and may jeopardize the long-term viability of wild populations. These are established facts—yet we must continue to repeat, again and again, that killing neck snares should be banned.

From 2010 to 2020, Canadian trappers captured at least 28,215 wolves [58]. This is a conservative estimate, as it excludes wolves that escaped and later died from their injuries [5]. Considering that fur trappers typically use killing neck snares to capture large carnivores [59], the majority of these wolves were likely snared. Even if I conservatively assume that only 60% were caught in snares, this means that 16,029 wolves were snared during that decade—an average of 1692 wolves per year, or about five wolves per day.

Snared wolves do not lose consciousness quickly. If I assume that each wolf suffered for at least one hour before losing consciousness, the cumulative amount of pain and distress on traplines equals ≥70 full days of suffering per year. If, as some evidence suggests, wolves may take ≥3 h to lose consciousness [13], the total amount of time spent in distress—struggling, chewing the snare, jumping, twisting, and collapsing—would equal 212 days per year. Despite this immense suffering, killing neck snares remain acceptable to the “relevant competent authorities” within provincial and territorial government agencies.

What is next?

As a scientist, I am convinced that killing neck snares must be banned. However, I cannot stop asking what must be performed to convince both the public and the scientific community to take meaningful action. This leads to several pressing questions:

- Shouldn’t killing neck snares be subject to the same criteria that are applied to other trapping devices used for the capture of large furbearers, namely canids?
- Given the fact that killing neck snares simply are inhumane restraining trapping devices, shouldn’t they be replaced with restraining devices that have been found to be humane?
- In the past, when the fur market was slow to drive innovation in trap technology, the threat of a trade embargo by the European Community led to the ban on steel-jawed leghold traps and the development of humane trapping standards [15]. Should a similar embargo from fur-buying countries be necessary to ban the use of neck snares?
- Previously, Stevens and Proulx demonstrated how proactive, persistent communication of scientific evidence to decision-makers, wildlife agencies, and the public led to the banning of an inhumane trapping device for northern raccoons (*Procyon lotor*) [60]. Should wildlife professionals and environmental organizations now launch national and international campaigns to raise awareness and pressure governments to ban killing neck snares?

Answering these questions—and acting on them—could lead to wildlife management programs that genuinely prioritize animal welfare and conservation.

## 5. Conclusions

The use of killing neck snares, which have been repeatedly recognized to be inhumane and non-selective, needs to be denounced. In societies that claim to be progressive and concerned with animal welfare, the fur market should not be allowed to benefit from cruelty and animal suffering. Major reforms are urgently needed in fur trapping practices, beginning with a ban on killing neck snares. All wildlife management programs and human activities involving mammal trapping should require that trapping devices meet the highest scientifically established animal welfare standards [1,17].
